# Bioimpedance assessment of body composition in the first adulthood period of somatic types residing in highland

**DOI:** 10.2478/joeb-2024-0014

**Published:** 2024-10-05

**Authors:** Kadyr Kozuev, Toktogazy Tulekeev, Sagynbek Dzholdubaev, Zarina Toichieva, Zhypargul Abdullaeva

**Affiliations:** 1 Department of Anatomy, Histology and Normal Physiology, International Medical Faculty, Osh State University, 723500, Osh, Kyrgyzstan; 2 Department of Human Anatomy and Morphology, Salymbekov University, 720054, Fuchik street 3, Bishkek, Kyrgyzstan

**Keywords:** First period of adulthood, men, bioimpedance assessment, somatic types, body mass composition, high altitude

## Abstract

The current stage of development of medical science is characterized by growing interest in constitutional typology and clinical anthropology. The anatomical-anthropological approach is an integrative technique of biology and medicine that allows us to determine the criteria for normality and pathology of a person. *Purpose of the study:* comparative bioimpedance assessment of somatic types of body component composition among the first mature age period people residing in highland. *Study design:* a comparative bioimpedance assessment of body types according to the Heath and Carter’s scheme was performed to identify somatic types and body weight composition in healthy men of the 1^st^ adulthood period living in high mountains (2469–3325 m above sea level). Somatotypical features of body composition have been established. An intertype and correlation analysis of body composition was conducted in individuals with different body types. *Results:* comparative bioimpedance and correlation analyses revealed differences in body composition indicators depending on population, age, body types and living conditions. *Conclusion:* mesoectomorphy, balanced ectomorphic, central and meso-endo types prevailed among the somatotype subgroups.

## Introduction

The morphological assessment of the individual somatic type, morphotype, genotype, body type serves as its cardinal basis and as relevant element in the biomedical research fields and daily practice in the medical and nutritional science; body composition can be interpreted and measured according to five organizational levels including variables measurement such as fat mass (FM), fat-free mass (FFM), total body water (TBW), bone mineral content, intra (ICW), and extra cellular (ECW) fluids, and different anthropometric measurements and index [1–4]. The anatomical variability of the somatic types of various ethnic groups and nationalities has not been sufficiently studied [5–8]. One of the common methods of somatotypology is Sheldon’s scheme, which modifies Heath and Carter’s applicability for both genders of all nationalities and races in a wide age range [9, 10]. The main indicator of nutritional status is the body mass component composition (muscle, bone, fat, etc.). Bioimpedance analysis, as an operational, non-invasive and highly informative method, allows one to assess the condition of protein, fat, water and mineral metabolism [11–18].

In the literature, there is a lack of data on the physical status of a healthy person, concerning different age-sex criteria [19,20]. The first period of adulthood is maturity itself, characterized by the end of the growth processes, the relative stability of definitive parameters [21] and it reflects the indicators of the young, able-bodied, reproductive strata of the population [22].

However, there is no information on the somatotypological characteristics of healthy people in the first adulthood period, considering the body component composition of highland residing people in the Kyrgyz Republic. A comparative bioimpedance assessment of somatotypes and body composition for people living in the high and low mountain conditions has been performed. Studies on body composition using bioimpedanosometry according to Heath and Carter’s body diagrams were conducted during the first adulthood period Kyrgyz people with various somatotypes residing in the highland [23–25].

In this study, 208 healthy men living in highlands aged from 22 to 35 years old and permanently residing at an altitude of 2469 – 3325 m above the sea level, were examined. For comparison, 221 men living in Osh city at an altitude of 870 - 1110 m above sea level were also examined.

The purpose of the study is to perform bioimpedance comparative assessment of somatic types and body component composition among the first mature age period of people residing in the highland.

The main research findings were obtained after analysis of body component composition, bioimpedanocemetry, correlation analysis and the index method.

## Materials and methods

### Anthropometry (somatometry)

Body length (BL) and body weight (BW) were measured using the Martin’s anthropometer (with an accuracy of 1 mm) and on an electronic scale (with an accuracy of 0.1 kg). We determined the length (cm), girth parameters (with a plastic tape, cm), and the thickness of the skin-fat folds (SFF) of different parts of the body with a caliper-compass (with an accuracy of 0.1 mm, contact surface area 90 mm^2^). Waist-to-hip ratios (W/H) were calculated. Body surface area (BSA) was determined by the Jackson’s formula (1958): S=100+W+(H-160) /100, where: S – BSA in m^2^, W – body weight, (r); H – body length, cm. A total of 27 anthropometric parameters were determined.

### The index method

Eight atomical indices of physical development and body composition were analyzed. Body mass index (BMI) – allowing to determine the correspondence between body weight and length: I = m/h2, m – body weight, kg; h – body length in square meters. WHO indices: weight-for-height (WFH); height-for-weight (HFW); weight-for-age (WFA) – indicators of malnutrition, short stature, and discrepancy between age and body weight.

### Bioimpedansometry

Bioimpedansometry was performed using an ABC-02 “Medass” body composition analyzer (NTS Medass, Russia) with a standard four-electrode circuit. Body types (12 dimensional characteristics) according to the Heath and Carter’s method were revealed after software calculation based on equations [10,11]. According to the Heath and Carter’s method, there are three main groups of somatotypes: mesomorphic (MM), ectomorphic (EcM), and endomorphic (EnM) divided into subgroups.

### Analysis of body composition

The body content main components were determined in absolute and relative values: fat mass (FM), kg, normalized by % FM; lean (without fat) mass (LM), kg; active cell mass (ACM), kg; % (share) of ACM; skeletal muscle mass (SMM), kg; % (share) SMM; total fluid (TF), kg; extracellular fluid (ECF), kg; phase angle (PA) in degrees.

Statistical processing was carried out using the R computing (version 4.2.1) in the RStudio development environment (version 2022.02.1+461). Additionally, R packages were used as psych, stats, dplyr, and car. Obtained results were visualized using the ggplot2 and ggcorrplot packages. Statistical relevance was considered when obtaining a P significance level of <0.05, in the case of multiple comparisons using the Mann-Whitney’s test at <0.0167.

### Correlation analysis

Relationship between parameters of body composition and body anthropometric indicators was established using Pearson’s tests (for normally distributed data with a linear nature of the relationship) and Spearman’s tests for data whose distribution differed from normal, or where the relationship was not linear.

### Informed consent

Informed consent has been obtained from all individuals included in this study.

### Ethical approval

This research including participation of human being has been complied with all relevant national regulations, institutional policies and in accordance with the Helsinki Declaration, and has been approved by the Osh State University Ethical Committee.

## Results

**Fig.1:**
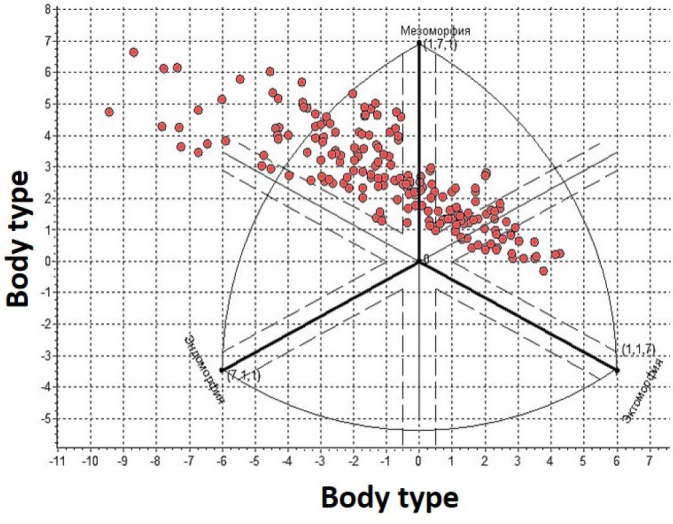
Distribution of somatotypes of highlanders according to Heath B.H. and Carter J.E.

Data analysis after somatometric and bioimpedance studies on males living in the highlands showed differences in the ratios of three main groups. A significant proportion of the highland residing population consisted of individuals with endomorphic 46.154% (n = 96) and ectomorphic 32.692% (n = 68) body types. Cohorts of individuals with mesomorphic somatotypes were registered in 21.154% (n = 44) of males living in the highland. Table 1 and Figure 1 show information on quantitative relationships and distributions of somatotypes.

**Table 1:** Indicators of subgroups of the main group of somatotypes according to Heath B.H., Carter J.E.

Somatotype		in %	n
Main group	Sub group		
Mesomorphic	Balanced mesomorphic	6.25	13
	Ectomesomorphic	4.807	10
	Meso – ecto	4.807	10
	Mesoectomorphic	16.827	35
Ectomorphic	Balanced ectomorphic	12.5	26
	Endoectomorphic	10.577	22
	Endo-ecto	2.885	6
	Ectoendomorphic	1.923	4
Endomorphic	Balanced endomorphic	3.365	7
	Mesoendomorphic	6.731	14
	Meso - endo	11.058	23
	Endomesomorphic	9.135	19
Central		9.135	19

From the MM cohort, mesoectomorphy predominates (16.827%, n = 35), EcM – balanced ectomorphy (12.5%, n = 26), EnM – meso-endo (11.058%, n = 23), and the central body type in 9.135% (n = 19) of men.

Comparison of the anthropometric parameters of mesomorphic (MM) males residing in highlands with similar data from males residing in low mountains shows that the body length of city residents is significantly higher P<0.05 (3.6 cm), while body mass (BM) and chest circumference (CC) are not significantly different. In males with ectomorphic somatotype, comparison of all body dimensions with city residents did not reveal significant differences (P<0.5). In males with endomorphic somatotype living in highlands, chest circumference predominated (P<0.05), (Table 2).

**Table 2:** Comparative data of overall parameters of men with different somatotypes in high and low altitudes.

Somatotype	Place	Parameter		
		BL (cm)	BM (kg)	CC (cm)
Mesomorphic	Highland	168.9±0.458	71.464±0.767	95.536±6.48
	Osh city	*172.07±0.447	70.57±0.67	94.46±0.421
Ectomorphic	Highland	174.134±0.378	62.082±0.44	88.474±0.408
	Osh city	174.74±0.397	61.578±0.46	84.84±0.326
Endomorphic	Highland	174.6±0.465	78.889±0.776	99.136±0.343
	Osh city	172.78±0.44	75.74±0.75	*96.32±0.48

1 *Note:* *P<0.05, compared with native highlanders. Numbers before ± symbol are mean values, after the symbol are errors of the mean.

The body surface area of the compared groups has not revealed significant differences. In men residing in the highland with mesomorphic somatotype, the body mass index (BMI) and waist-hip (W/H) ratios were predominated (Table 3). In endomorphic somatotypes, the W/H ratio is 1.82 times higher compared to the same indicators of city residents.

**Table 3:** Comparative data of BSA, BMI and W/H ratio in men of different somatotypes from high and low altitudes.

Somatotype	Place	Parameter		
		BSA (m^2^)	BMI (kg/m^2^)	W/H
Mesomorphic	Highland	1.804±0.011	24.978±0.22	0.9 ±0.004
	Osh city	1.82±0.01	*23.6±0.146	0.871±0.003
Ectomorphic	Highland	1.762±0.008	20.436±0.093	0.855±0.003
	Osh city	1.763±0.008	20.15±0.102	0.856±0.003
Endomorphic	Highland	1.936±0.011	25.777±0.19	0.918±0.003
	Osh city	1.85±0.01	25.32±0.21	*0.902±0.003

1 *Note:* *P<0.05, compared with native highlanders. Numbers before ± symbol are mean values, after error of the mean.

## Discussion

Bioimpedansometry revealed somototypological features of the body component composition parameters. Men living in highlands with endomorphic somatotype have a high content of absolute and relative active cell mass (ACM) and phase angle (PA) (P<0.05 – 0.01), with a low percentage of skeletal muscle mass (SMM). In 75% of EnM men living in highlands, the phase angle is increased by 1.12 times (8.3°, P<0.05), which indicates a high level of training and endurance [10, 17].

In men with endomorphic somatotype residing in the Osh city low mountainous area, there was an increase in the values of HFW (1.1 times), predominance of the skin fat fold (SFF) thickness in the shoulder area at the back, above the iliac crest and lower leg (P<0.01), with a relatively low value of weight, body length and body surface area (BSA). BMI in EnM for Osh city residents significantly exceeds the normal value (25.67–29.82 kg/m^2^), and 75% of people are diagnosed with excess body weight.

In males with ectomorphic somatotype living in the Osh city, in comparison with the highland ectomorphic somatotypes, the SMM predominated (1.03 and 1.028 times), HFW and WFA (1.07 and 1.06 times) predominated, and low values of PA by 0.71° (7.409±0.51), P<0.05 were found. In the highland residing men with ectomorphic somatotype, there are significant increases in the absolute and percentage contents of FM, ACM, BFM, active cell mass index (ACMI) and PA. The thickness of the SFF of the chest and anterior abdominal wall prevails p<0.05.

In men with mesomorphic somatotype in Osh, HFW and WFA were predominated. The phase angle exceeds normal values (7.99±0.056°, 8.6°, 3^rd^ quartile). Low FM content and % ACM content, P<0.05.

**Table 4:** Comparative data of bioimpedance measurement in men of different somatotypes from high and low altitudes.

Somatotype	Place	Parameter			
		FM (kg, %)	LM (kg)	ACM (kg, %)	SMM (kg, %)
Mesomorphic	Highland	14.62±0.466 19.707%	56.843±0.429	36.861±0.312 64.772%	29.99 ±0.241 52.743%
	Osh city	12.6±0.363 16.60%	58.28±0.38	36.14±0.32 *62.27%	*31.61±0.202 *54,26%
Ectomorphic	Highland	9.1±0.236 14.36%	52.963±0.281	33.253 ±0.222 62.737%	28.794±0.159 54.388%
	Osh city	*7.747±0.228 *12.22%	53.919±0.319	*32.305±0.226 *59.879%	*29.827±0.183 *55.304%
Endomorphic	Highland	19.011±0.499 23.648%	59.807±0.457	38.702±0.316 64.634%	31.186±0.264 52.105%
	Osh city	17.13±0.43 22.06%	58.42±0.37	*35.72±0.26 *61.13%	30.88±0.18 *52.91%

1 *Note:* *P<0.05, compared with native highlanders. Numbers before the ± symbol are mean values, after the symbol are errors of the mean.

**Table 5:** Comparative data on the phase angle, total and extracellular fluid in men of various somatotypes from high and low altitudes.

Somatotype	Place	Parameter		
		Total liquid (kg)	ECF (kg)	Phase angle (°)
Mesomorphic	Highland	41.607±0.314	16.187±0.132	8.681±0.046
	Osh city	42.66±0.283	16.68±0.119	*7.99±0.056
Ectomorphic	Highland	38.764±0.207	15.141±0.08	8.117±0.043
	Osh city	39.47±0.234	15.454±0.098	*7.409±0.051
Endomorphic	Highland	43.78±0.334	17.118±0.144	8.657±0.041
	Osh city	42.8 ±0.27	16.75±0.11	*7.69±0.05

1 *Note:* *P<0.05, compared with native highlanders. Numbers before ± symbol are mean values, after the symbol are errors of the mean.

Men residing in highlands with mesomorpic somatotype have low SMM values, with high values of IFM, ACMI 1.26 and 1.05 times, BMI and PA 8.681±0.046°, 9.2°, 3^rd^ quartile, P<0.01. The majority of MM highland residing men (75%) have excess body weight, BMI is 25.9 kg/m^2^, median, and the predominance of the abdominal wall thickness, the front shoulder 1.11 times and the anterior abdominal wall 1.24 times, 22 mm, 3^rd^ quartile.

Correlation analysis revealed variability in the strength and degree of relationship between the compared values. Positive correlations are often found between the variables: MT - DT; BMI – FM, %FM; OGK – waist circumference; ZhM – %ZhM; TM – ACM, SMM, VKZh; ACM – %ACM, OZH, VKZH; PA – %ACM, ACM; Coolant – VKZh (r = >0.7).

Negative – BMI – %SMM (r = - 0.73); ZhM – %SMM (r = - 0.81); TM – %FM (r = - 0.63); %FM – %SMM (r = - 0.85); %ACM – BMI (r = - 0.62).

## Conclusion

Comparison of frequency in the main male somatotypes living in the highland and low mountains revealed differences in the frequency distributions of subtypes: increase in meso-endomorphic somatotype (16.29%), central form (12.67%) and ectoendomorphic somatotype (9.50%), with decrease in the proportion of mesoectomorphic somatotype to 8.6% (low mountains). Comparative bioimpedance and correlation analysis established differences in body composition indicators depending on populations, age, body types and living conditions. Low variation between study subjects in the body mass shown in table 2 may be due to lifestyle and same environmental conditions.
